# Modelling the formation and self-healing of creep damage in iron-based alloys

**DOI:** 10.1007/s10853-018-2666-9

**Published:** 2018-07-17

**Authors:** C. D. Versteylen, M. H. F. Sluiter, N. H. van Dijk

**Affiliations:** 1 Fundamental Aspects of Materials and Energy, Faculty of Applied Sciences, Delft University of Technology, Mekelweg 15, 2629 JB Delft, The Netherlands; 2 Virtual Materials and Mechanics, Department of Materials Science and Engineering, Delft University of Technology, Mekelweg 2, 2628 CD Delft, The Netherlands

## Abstract

A self-consistent model is applied to predict the creep cavity growth and strain rates in metals from the perspective of self-healing. In this model, the creep cavity growth rate is intricately linked to the strain rate. The self-healing process causes precipitates to grow inside creep cavities. Due to the Kirkendall effect, a diffusional flux of vacancies is induced in the direction away from the creep cavity during this selective self-healing precipitation. This process impedes the creep cavity growth. The critical stress for self-healing can be derived, and an analysis is made of the efficiency of self-healing elements in binary Fe–Cu, Fe–Au, Fe–Mo, and Fe–W alloys. Fe–Au is found to be the most efficient self-healing alloy. Fe–Mo and Fe–W alloys provide good alternatives that have the potential to be employed at high temperatures.

## Introduction

High-temperature deformation and failure mechanisms in metals have attracted considerable academic and industrial attention since the 1950s [1–4]. The materials’ response can be complicated since many processes are at play simultaneously, such as dislocation glide and climb, jog and wall formation, vacancy formation and annihilation, and creep cavity nucleation and growth. Sandström and co-workers developed models which can predict creep rates, based on the formation and annihilation rates of dislocations. These models can provide accurate predictions of the creep rates of various alloys [5–7]. A key damage mechanism is the nucleation of creep cavities located at the grain boundaries oriented perpendicular to the applied load at elevated temperatures [8].

After nucleation, these cavities start to grow by the diffusion of vacancies [9] and eventually they coalesce with neighbouring cavities formed on the same grain boundary. Taking into account the formation of the creep cavities as well as the macroscopic strain rate is a difficult task which can be done by taking a cohesive zone model [10], or by establishing a link with strain rate and creep void growth rate through the model by Sandström [7, 11]. After coalescence, a rapid damage growth is observed, resulting in macroscopic failure. In this failure mechanism, the creep time is inversely proportional to the creep strain rate of the alloy. This behaviour is known as the Monkman–Grant relation. Linking the strain rate to the cavity growth has been a key subject of interest [12–21].

Recently, the concept of self-healing has been explored to extend the lifetime of structural and functional man-made materials [22]. Autonomous repair of creep damage has been investigated by Laha and co-workers for stainless steels [23, 24] and by Zhang and co-workers for ferritic Fe–Au [25–28] and Fe–Mo alloys [29]. In these studies, solute elements are brought in a supersaturated state and thereby show a strong tendency to segregate. It was found that up to 80% of the creep damage could be filled by selective precipitation growth at creep cavity surfaces [26]. This autonomous repair mechanism is demonstrated to significantly extend the lifetime and thereby lead to a more creep-resistant metal. The current status of these and other approaches for self-healing alloys are reviewed by van Dijk and van der Zwaag [30]. Where creep failure is largely controlled by the diffusion of vacancies, the self-healing of creep damage largely relies on the diffusion of supersaturated solute. This means that self-healing of creep damage requires a new theoretical framework to describe creep damage and healing, based on a delicate balance between the simultaneous diffusion of host atoms (vacancies) and solute atoms. The aim of this work is to link the transport mechanisms of excess vacancies, supersaturated solute, and the macroscopic strain rate in creep-healing high-temperature metal alloys. Recently, we proposed a conceptual model based on the transport of vacancies between bulk, grain boundary, and creep cavities [31]. In the present paper, these ideas are applied to formulate a mathematical model to quantitatively predict the formation and self-healing of creep damage in iron-based alloys. This quantitative model has been used to evaluate critical stress (as a function of temperature) below which self-healing is possible. The creep behaviour of the extensively studied binary Fe–Au (1 at.%) alloy [25–27] is used as an example to optimise the temperature and stress dependence for the healing of creep damage. The healing potential of Fe–Au alloys is compared to that of Fe–Cu, Fe–Mo, and Fe–W alloys.

## Model description

### Constrained growth of creep cavities

**Figure 1:**
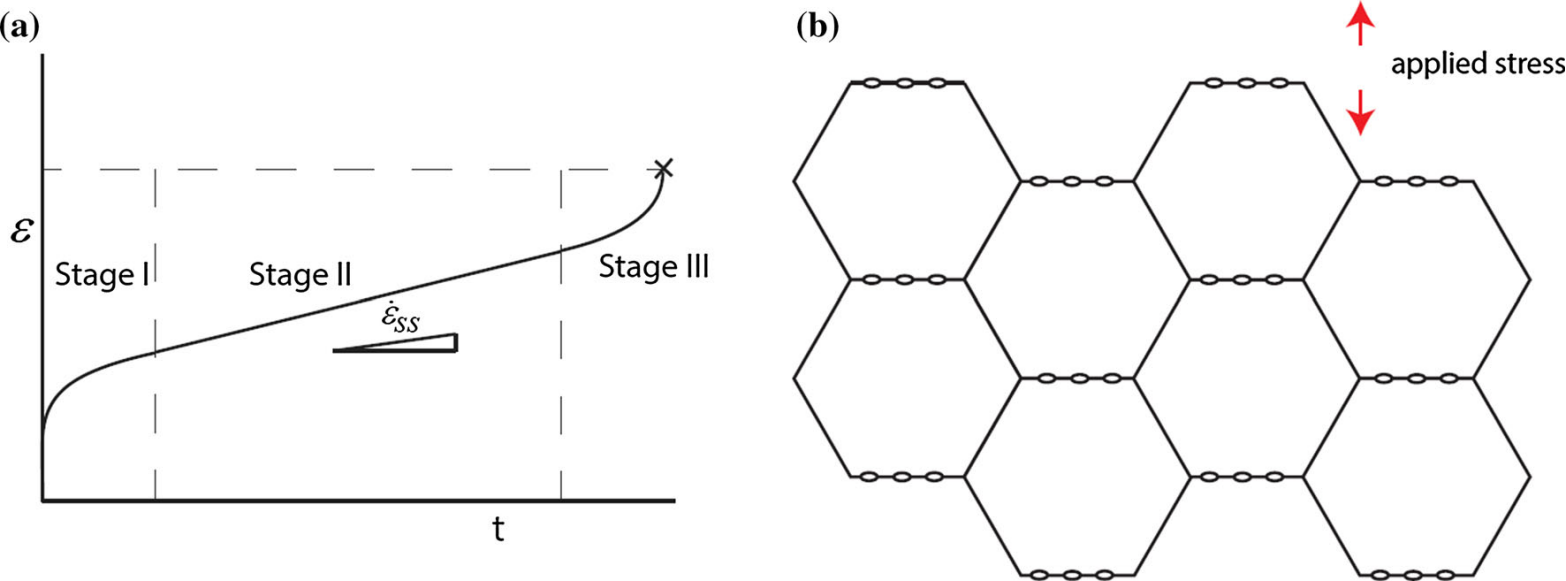
**a** Strain–time curve of a metal under creep conditions. In stage II, a steady-state strain rate $\left( \dot{\epsilon }_{\mathrm{ss}}\right)$ is observed. **b** Formation of creep cavities at grain boundaries oriented perpendicular to the applied stress direction

**Figure 2:**
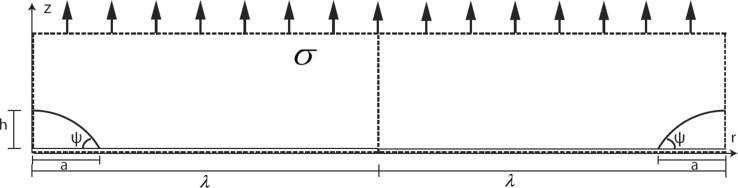
Creep cavity of width 2*a* on a grain boundary in a metal under a stress $\sigma$, where the distance $2 \lambda$ indicates the distance to the next creep cavity. The creep cavities are lens-shaped with a height *h* and an opening angle $\psi$

As is shown in Fig. 1a, a creeping material generally deforms in three stages: an initial stage when load and temperature are first applied (stage I), a steady-state constant creep rate (stage II), and finally an accelerated creep rate until failure (stage III). Under the influence of stress, creep cavities form at grain boundaries oriented perpendicular to the stress direction, as visualised in Fig. 1b. The geometry of the stress affected grain boundary with two neighbouring creep cavities is illustrated in Fig. 2.

The damage formation in creeping metals was first described by Hull and Rimmer [9]. In this model, creep cavities form on grain boundaries and grow through the diffusional flux of vacancies, which is driven by a gradient in chemical potential of a vacancy between a location far away from the creep cavity and at the cavity surface. The applied stress $\sigma$ causes an effective stress $\sigma _b$, on the grain boundary far away from the cavity, and the stress which results from the surface energy of the creep void $\sigma _0$ on the tip of the cavity. For a fraction $\omega$ of cavitated grain boundary surface, the gradient in the chemical potential is [4] (Fig. 2).

1 $$\begin{aligned} \left| {\vec {\nabla }} \mu \right| = \frac{8 \pi \sigma _b - (1-\omega ) \sigma _0}{(-2 \ln (\omega ) - (3-\omega ) (1-\omega ))}. \end{aligned}$$

The gradient of the chemical potential strongly depends on $\omega$. For a small $\omega$, and $\sigma _0~<<~\sigma _b$, the effective chemical potential gradient is close to the original prediction by Herring [1] and Hull and Rimmer [9]. The gradient in chemical potential $\nabla \mu$ can then be approximated by:

2 $$\begin{aligned} \left| \vec {\nabla } \mu \right| \approx \frac{\sigma \varOmega }{\lambda }. \end{aligned}$$

Stress $\sigma$ acting on a vacancy with volume $\varOmega$ causes an increase in its chemical potential. At the edge of the creep cavity, this stress is effectively zero and far away from the cavity; at distance $\lambda$, it is equal to the applied stress. This stress gradient results in a gradient in the chemical potential for a vacancy. The cavity grows due to a diffusional vacancy flux over grain boundaries (vacancies are indicated by the symbol $\square$);

3 $$\begin{aligned} J_{\square } = -\frac{1}{\varOmega }\frac{D_{\square }^{\mathrm{gb}} x_{\square }^{\mathrm{gb}}}{k_{\mathrm{B}}T} \nabla \mu \approx -\frac{D_{\square }^{\mathrm{gb}} x_{\square }^{\mathrm{gb}}}{k_{\mathrm{B}}T} \frac{\sigma }{\lambda }, \end{aligned}$$

where $k_{\mathrm{B}}$ is Boltzmann’s constant and *T* is the temperature. The flux of vacancies towards the creep cavity $J_{\square }$ is a function of the vacancy diffusivity over the grain boundary $D_{\square }^{\mathrm{gb}}$ (which is much faster than bulk diffusivity $D_{\square }^{\mathrm{bulk}}$) and a function of the equilibrium vacancy concentration at the grain boundary, $x_{\square }^{\mathrm{gb}}$ (in mole fraction). This approach of Hull and Rimmer [9] provides a diffusional flux, driven only by applied stress (they introduced a vacancy density $C_{\square }^{\mathrm{gb}} = x_{\square }^{\mathrm{gb}} / \varOmega$). This flux contributes to the growth of the creep cavity by adding a volume $\varOmega$ for each added vacancy. The creep cavity surface connecting the grain boundary is equal to $S=2\pi a \delta$, where *a* is half the opening width of the creep cavity and $\delta$ is the grain boundary thickness.

The void growth rate $\dot{V}= -J_{\square }S\varOmega$ can now be described as:

4 $$\begin{aligned} \dot{V} = \frac{D_{\square }^{\mathrm{gb}} x_{\square }^{\mathrm{gb}}}{k_{\mathrm{B}} T} \frac{\sigma }{\lambda } 2\pi a \delta \varOmega . \end{aligned}$$

During typical operating conditions, creep deformation is dominated by steady-state creep, also known as stage II creep (see Fig. 1). For these conditions, the time to failure $t_f$ depends directly on the steady-state strain rate $\dot{\epsilon }_{\mathrm{ss}}$. This is known as the Monkman–Grant relation [32]:

5 $$\begin{aligned} \dot{\epsilon }_{\mathrm{ss}} t_f = C_{\mathrm{MG}}, \end{aligned}$$

where $C_{\mathrm{MG}}$ is the Monkman–Grant constant. At first glance, Eqs. 4 and 5 seem to contradict each other since $\dot{\epsilon }_{\mathrm{ss}}$ is normally related to the bulk diffusivity and the void growth rate $\dot{V}$ to the grain boundary diffusivity.

The description of creep has been divided into microscopic damage descriptions [9, 33] and the macroscopic strain rate description, i.e. the Monkman–Grant relationship [4, 32]. In order to explain the relation between this large-scale deformation model and the small-scale damage model, the principle of constrained creep cavity growth was proposed. This constrained growth was first introduced by Ishida and McLean [12] to explain discrepancies between theoretical unconstrained cavity growth and experimentally observed creep curves. In their approach, a grain boundary requires the ingress of a dislocation to form a vacancy. The steady-state creep strain rate $\dot{\epsilon }_{\mathrm{ss}}$ is thereby coupled to the volumetric growth rate $\dot{V}$ of a creep cavity.

The link between the steady-state strain rate $\dot{\epsilon }_{\mathrm{ss}}$ and the cavity growth rate $\dot{V}$ leads to the Monkman–Grant relation. Dyson [13] showed that in many cases the creep cavity growth rate will be limited by the strain rate of the material;

6 $$\begin{aligned} \dot{\epsilon }_{\mathrm{ss}} = \frac{\dot{V}}{4 \lambda ^2d}, \end{aligned}$$

where *d* is the grain size and $4\lambda ^2$ is the grain boundary surface area assigned to a single cavity.

Building on the ideas of Dyson, Rice [19] formulated a model where the rate of opening for creep cavities is a function of the strain rate by combining both effects. This was worked out by Needleman and Rice [20] and Budiansky [34]. Van der Giessen and co-workers [21] analysed this effect for different applied load combinations. However, these studies do not treat the case where the strain rate is a limiting factor on the diffusional growth rate of a cavity, which is treated here. Cocks and Ashby [35] reviewed all different creep regimes and provided maps of the damage rate as a function of applied stress. Similar to the model of Rice [19], Riedel developed a model which links the strain rate with void growth rates [4] and very recently this was extended by Sandström [11]. In all these models, the growth of creep cavities and the strain rate are linked to the constrained growth. These descriptions can provide good agreement with experimental data for conventional creep, but they do not describe self-healing systems. It also does not provide an explanation why the strain rate and void growth rates are linked.

We follow the ideas of Ishida and McLean and assume that the ingress of a dislocation to the grain boundary can cause the formation of a vacancy, which in turn contributes to the growth of a creep cavity. If these vacancies are not formed continuously, the void growth rate would come to a stop. Thereby the growth rate of creep cavities and the creep strain rate are linked through the movement of dislocations, where the rate-limiting step is the dislocation climb in the bulk (leading to an activation energy similar to the self-diffusion activation energy in the bulk).

The assumptions used in the proposed model are:

1. Creep cavities form at grain boundaries perpendicular to the loading direction.
2. Cavity growth rate and the steady-state strain rate are proportional.
3. Continuous formation of vacancies is required in order to maintain the cavity growth rate, and these vacancies form predominantly as a result of dislocation ingress at grain boundaries. This means that the vacancy formation is the rate-limiting step for the diffusional growth of creep cavities.
4. The ingress of dislocations to a grain boundary can cause excess volume and stress concentrations to accumulate in the grain boundary. The relaxation of the excess volume and the stress concentration on the grain boundary can happen by draining vacancies from the grain boundary to the creep cavities.
5. Supersaturated solute has a preference for precipitation at the creep cavity surface.

### Dislocation movement and vacancy transport

In the present models that describe creep cavity growth [9], the implicit assumption is that the vacancy concentration remains at equilibrium values at a characteristic distance from the creep cavity at all times. It is not a priori obvious that this should be true. In fact, the concepts of Ishida and co-workers [12, 36] that a grain boundary requires the ingress of dislocations in order to be able to slide can be combined with the proposal of Dyson [13, 37] that grain boundary sliding is a constraint for the growth of creep cavities.

This means that the movement of dislocations, which controls the strain rate of metal that deforms under creep conditions, is also the rate-determining factor for cavity growth. This sheds some light on the Monkman–Grant relationship: the strain rate determines the time to failure by the formation of vacancies on the grain boundaries close to the diffusion zone of the creep cavities.

When a dislocation network has developed and the steady-state strain rate causes a certain number of dislocations per second to reach a grain boundary, each of them carries an open volume [38], part of which is transferred to the grain boundary when the dislocation impinges. The vacancy fraction in the grain boundary $x_{\square }^{\mathrm{gb}}$ should depend on the rate at which vacancies are generated due to the influx of dislocations.

For climb-controlled creep, the strain rate of a metal depends on the mobile dislocation density. Using the Orowan equation for these cases [39], with the dislocation density $\rho _{\mathrm{disl}}$, the climb velocity $v_{\mathrm{cl}}$ of a dislocation jog and the Burgers vector *b*,

7 $$\begin{aligned} \dot{\epsilon } = b \rho _{\mathrm{disl}} v_{\mathrm{cl}}. \end{aligned}$$

The strain rate depends on the stress through the dislocation density [40] and the climb velocity [41]. The stress dependence of the strain rate is expressed with a power law as, $\dot{\epsilon }\propto \sigma ^n$.

The stress dependence of the dislocation climb velocity [42] can be approximated by:

8 $$\begin{aligned} v_{\mathrm{cl}} \approx \frac{D_{\mathrm{sd}} f_{\mathrm{cl}} \varOmega }{b^2 k_{\mathrm{B}}T}, \end{aligned}$$

where $D_{\mathrm{sd}}$ is the iron self-diffusivity and $f_{\mathrm{cl}}$ the force acting on a climbing dislocation. During stage II creep with a constant strain rate, the average collective dislocation movement is of interest for the deformation rate. The drift velocity of the dislocation network can be correlated with the individual movements of dislocations [43]. The collective climbing or gliding rate of dislocations in a dislocation network is unknown, but as an approximation the individual movement can be considered. The strain rate according to the Orowan equation (Eq. 7) can be linked to the Dyson equation (Eq. 6), in order to obtain an equation of the creep cavity growth:

9 $$\begin{aligned} \dot{\epsilon } = b \rho _{\mathrm{disl}} v_{\mathrm{cl}} = \frac{\dot{V}}{4 \lambda ^2d}. \end{aligned}$$

The creep cavity growth rate $\dot{V}$ now depends on the influx of dislocations and the volume associated with these dislocations. The density of dislocations transported to the grain boundary is associated with the creep void by length $\lambda$. The dislocation density is a function of the subgrain size [44], for an observed subgrain size $\left( d_{\mathrm{sub}}\right)$ of 1 $\upmu$m in Fe–Au [25], the dislocation density $\rho _{\mathrm{disl}}~=~1\times 10^{-12}~{\hbox {m}}^{-2}$. For Fe–Au, it was found experimentally [25–27] that at 550° and an applied load of 100 MPa, the strain rate $\dot{\epsilon }=2\times ~10^{-8}~{\hbox {s}}^{-1}$. The Burgers vector of bcc iron $b = 2.5$ Å. The climbing velocity of the collective dislocation network then $v_{\mathrm{cl}}=8\times 10^{-11}~{\hbox {m~s}}^{-1}$. The associated velocity of the dislocation network is approximately 1 Å/s. This value is of similar magnitude compared to the values found by Caillard for single dislocation kink movement, in the presence of solute [41].

When a dislocation impinges on or near a grain boundary, it will provide a back stress on the following dislocations. The character of a grain boundary is altered by the absorption of a dislocation and its associated volume [45, 46]. This change in character, in the form of a stress concentration, provides a repulsive barrier for the influx of the next dislocation [47]. The increase in volume in the grain boundary leads to a more disordered structure and an excess vacancy concentration. It has been observed that the formation and growth of creep cavities are highly dependent on the grain boundary character of the surrounding grain boundaries [48, 49]. We postulate that the relaxation of the excess volume and the stress concentration on certain grain boundaries can happen by draining vacancies from these grain boundary to the creep cavities. This flux of vacancies from a disordered section of grain boundary to the creep cavities leads to a less disordered grain boundary and allows new dislocation to ingress into the grain boundary. This link of the grain deformation rate and the creep cavity growth rate causes the Monkman–Grant relation.

### Self-healing

**Figure 3:**
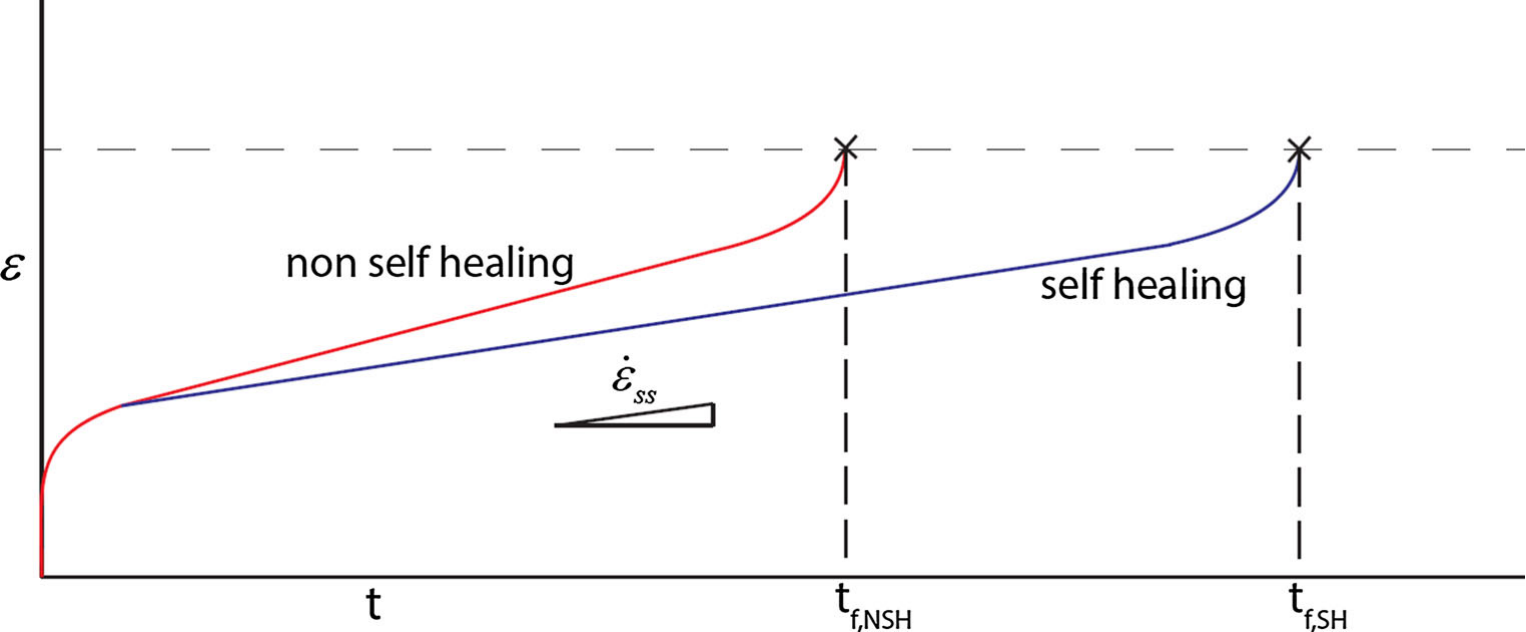
Evolution of strain with time for a non-self-healing and a self-healing alloy. The non-self-healing alloy has a shorter creep lifetime $t_{f,{\mathrm{NSH}}}$ and a higher steady-state strain rate $\dot{\epsilon }_{\mathrm{ss}}$. The time to failure is predominantly controlled by the strain rate in stage II

Experimentally, it has been observed that the presence of supersaturated solute can result in an autonomous filling of creep cavities and a significant extension of the creep lifetime [25–27]. It is found that the self-healing mechanism does not significantly affect the critical strain at rupture, but does reduce the steady-state strain rate, as schematically illustrated in Fig. 3. The solute that segregates at the free creep cavity surfaces is found to be transported along the grain boundaries from the supersaturated bulk. This flux of segregating solute competes with the vacancy flux and thereby reduces both the cavity growth rate and the vacancy flux away from grain boundaries under stress towards the creep cavities. This process is known as the Kirkendall effect.

**Figure 4:**
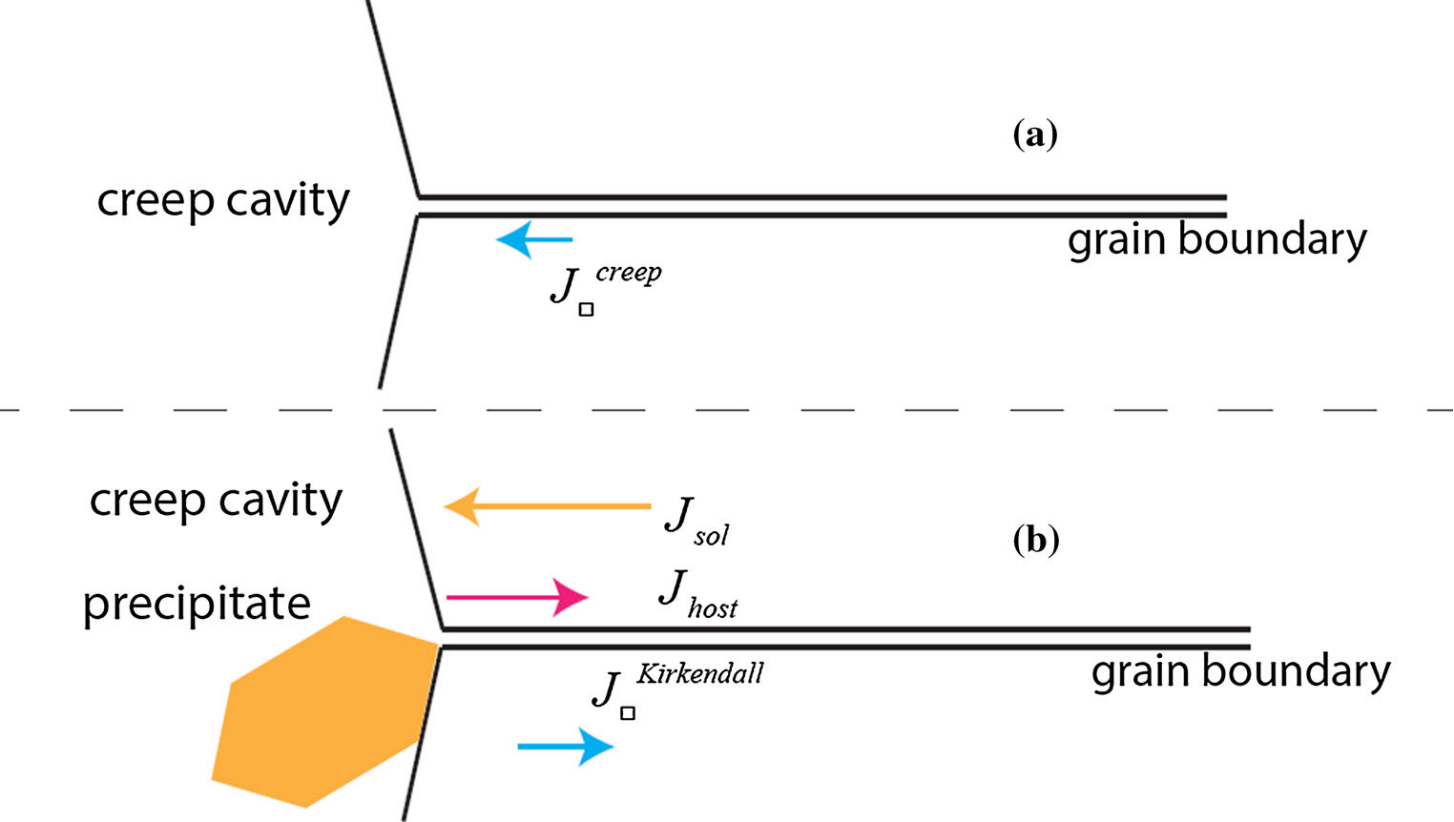
**a** Schematic illustration of the vacancy transport near a creep cavity. The flux of vacancies through a grain boundary towards a creep cavity during stage II creep causes this creep cavity to grow. **b** When precipitation occurs inside the creep cavity, a solute flux causes the precipitate to grow. The solute flux $\left( J_{\mathrm{sol}}\right)$ causes a vacancy flux $\left( J_{\square }\right)$ in the opposite direction due to the Kirkendall effect. The net vacancy flux can be zero, preventing the creep cavity to grow

### Solute transport

After nucleation, a diffusional growth of the precipitate initiates a flux of solute, driven by a chemical potential;

10 $$\begin{aligned} \vec {J}_{\mathrm{sol}} = -\frac{D_{\mathrm{sol}}}{k_{\mathrm{B}}T} \vec {\nabla } \mu _{\mathrm{sol}}. \end{aligned}$$

The difference in chemical potential of solute atoms between precipitation in the bulk and on creep cavity surface causes a preference for precipitate growth in the creep cavities. The terms playing a role are the possibility for the precipitate to reduce the surface energy of the free surface of bulk material in the creep cavity, the possibility of reducing the surface energy of a precipitate, and the reduction in stress concentration between the precipitate and the bulk material. The driving force for precipitation is then given by this chemical potential, but also by the supersaturated solute which remains in solution during service life. This is assumed to be the largest contribution to the self-healing process in metals, and it is measurable with atom probe tomography [25]; the solute is then depleted from the grain boundary and neighbouring bulk as a result of the diffusion towards the precipitate (Fig. 4).

The difference in diffusivity of host and substitutional solute causes a net diffusion of vacancies in the direction opposite to the faster species. The flux balance of this process can be approximated with the following Darken equation which, in the dilute limit, can be simplified to:

11 $$\begin{aligned} \vec {J}_{\square ,{\mathrm{out}}} = \frac{1}{\varOmega }(D_{\mathrm{sol}} - D_{\mathrm{host}}) \vec {\nabla } x_{\mathrm{sol}}. \end{aligned}$$

where $D_{\mathrm{host}}$ is the diffusivity of the host atoms, $D_{\mathrm{sol}}$ is the diffusivity of the solute, and $\nabla x_{\mathrm{sol}}$ is the concentration gradient of the solute. Assuming that the supersaturated solute $\Delta x_{\mathrm{sol}}^{\mathrm{gb}}$ shows a concentration profile over the grain boundary with a characteristic length $\lambda$, the gradient can be approximated by $|\vec {\nabla }| x_{\mathrm{sol}}=\Delta x_{\mathrm{sol}}^{\mathrm{gb}} /\lambda$. This approximation is valid in the dilute limit, with negligible off-diagonal terms of the Onsager matrix [50].

### Flux balance and critical stress

The opposite vacancy fluxes caused by the gradient in stress-induced chemical potential and by the solute gradient result in a net vacancy flux, either towards or from the cavity. Self-healing can be achieved when

12 $$\begin{aligned} J_{\square ,{\mathrm{out}}}^{\mathrm{gb}} \ge J_{\square ,{\mathrm{in}}}^{\mathrm{gb}}, \end{aligned}$$

where the flux of vacancies over the grain boundary towards the creep cavity $J_{\square ,{\mathrm{in}}}$ has to be smaller (or equal) than the flux of vacancies in the opposite direction $J_{\square ,{\mathrm{out}}}$. As discussed in the “Appendix”, the outflux of vacancies from the creep cavity is in most cases controlled by the diffusivity of solute through the bulk.

When the two fluxes are equal, a critical stress can be defined below which diffusional creep can be self-healed. Combining Eqs. 3 and 11, with $2\pi a \delta J_{\square ,{\mathrm{out}}}^{\mathrm{gb}} = 8 \lambda ^2 J_{\square ,{\mathrm{out}}}^{\mathrm{bulk}}$ (see “Appendix”), the flux balance results in the critical stress for self-healing,

13 $$\begin{aligned} \sigma _{\mathrm{crit}}= \frac{k_{\mathrm{B}}T}{\varOmega } \frac{4\lambda ^3}{\pi a \delta l} \frac{\left( D_{\mathrm{sol}}^{\mathrm{bulk}}-D_{\mathrm{host}}^{\mathrm{bulk}}\right) \Delta x_{\mathrm{sol}}^{\mathrm{bulk}}}{D_{\square }^{\mathrm{gb}} x_{\square }^{\mathrm{gb}}} . \end{aligned}$$

The critical stress for self-healing $\left( \sigma _{\mathrm{crit}}\right)$ depends on the solute diffusivity compared to the host diffusivity $\left( D_{\mathrm{sol}}^{\mathrm{bulk}}-D_{\mathrm{host}}^{\mathrm{bulk}}\right)$, the grain boundary diffusivity $\left( D_{\square }^{\mathrm{gb}}\right)$, the supersaturated solute concentration $\left( \Delta x_{\mathrm{sol}}\right)$, and vacancy concentration $\left( x_{\square }^{\mathrm{gb}}\right)$. The length *l* is the diffusion length of the supersaturated solute in the bulk towards the grain boundary. The maximum distance $\left( l_{\mathrm{max}} = \frac{\pi }{3} \frac{a^3}{\lambda ^2 \Delta c_0 \varOmega }\right)$ can be estimated from mass conservation (see “Appendix”).

### Cavity growth rate

Creep cavity growth rate can be estimated from the net vacancy flux integrated over the creep void area connecting the grain boundary:

14 $$\begin{aligned} \dot{V} = 2 \pi a \delta J_{\square }^{\mathrm{gb}} \varOmega . \end{aligned}$$

The rate-limiting factor for the void growth is the formation of vacancies, which is linked to the strain rate. The solute precipitation in the cavity is quickly limited by the bulk diffusional flux to the area surrounding the creep cavity $\left( 4\lambda ^2\right)$, see “Appendix”. For stage II creep where the supersaturated solute is transported exclusively to the creep cavities, it is possible to write the constrained cavity growth rate as:

15 $$\begin{aligned} \dot{V}= 2 \pi a \delta \frac{D_{\square }^{\mathrm{gb}} x_{\square }^{\mathrm{gb}}}{k_{\mathrm{B}}T} \frac{\varOmega \sigma }{\lambda } -8\lambda ^2 \left( D_{\mathrm{sol}}^{\mathrm{bulk}}-D_{\mathrm{host}}^{\mathrm{bulk}}\right) \frac{\Delta x_{\mathrm{sol}}^{\mathrm{bulk}}}{l} . \end{aligned}$$

The depletion of supersaturated solute from the bulk close to the grain boundaries is clearly observed by Zhang and co-workers [25]. This depleted zone points to a diffusion-controlled process. This proves that grain boundary sliding is not rate-limiting to the deformation.

Using Eq. 9, the strain rate of self-healing creep steels can be formulated as:

16 $$\begin{aligned} \dot{\epsilon }_{\mathrm{ss}}= \frac{\pi a \delta }{2\lambda ^2 d} \frac{D_{\square }^{\mathrm{gb}} x_{\square }^{\mathrm{gb}}}{k_{\mathrm{B}}T} \frac{ \varOmega \sigma }{\lambda } -\frac{2}{d}\left( D_{\mathrm{sol}}^{\mathrm{bulk}}-D_{\mathrm{host}}^{\mathrm{bulk}}\right) \frac{\Delta x_{\mathrm{sol}}^{\mathrm{bulk}}}{l} \end{aligned}$$

## Model predictions

The model is applied to the critical stress for the experimentally studied binary alloys: Fe–1at.%Cu and Fe–1at.%Au alloys [25–27].

**Figure 5:**
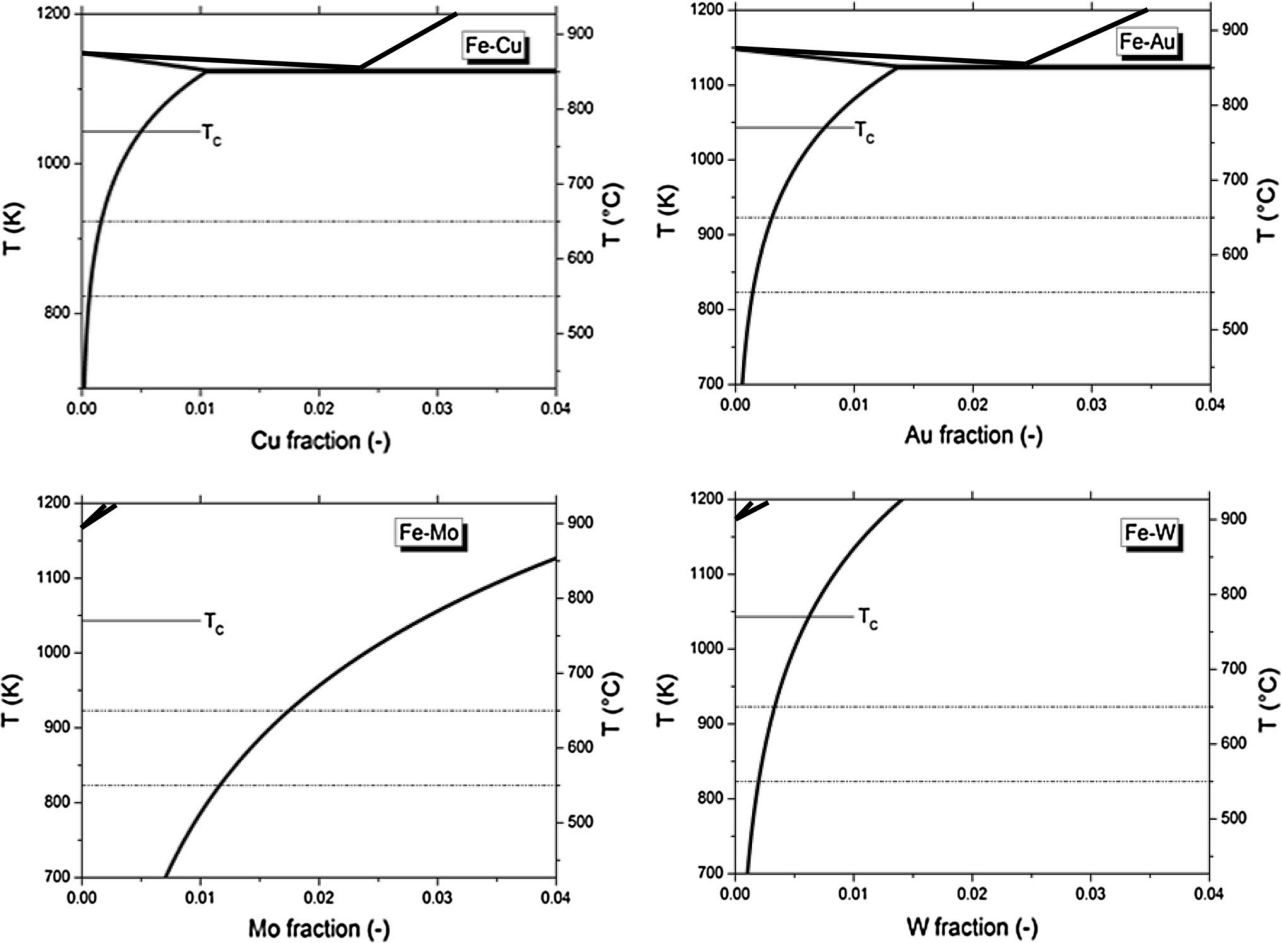
Partial phase diagrams for Fe–Cu, Fe–Au, Fe–Mo, and Fe–W binary alloys. With the creep temperatures 550° and 650° indicated as dashed lines. The magnetic Curie temperature ($T_{\mathrm{C}}$) of pure iron is indicated for reference. All fractions indicated in the figure are in at.%

The solubility of copper in bcc iron is obtained from Chen and co-workers [51], the solubility of gold in bcc iron from Okamota and co-workers [52], the molybdenum solubility [53], and tungsten solubility from Landolt–Börnstein [54]. The relevant part of the phase diagram (between 700 and 1400 K and between 0 and 4 at.% atom fraction of impurity) is presented in Fig. 5 for Fe–Cu, Fe–Au, Fe–Mo, and Fe–W binary alloys.

The grain boundary self-diffusivity of iron was measured over a wide temperature range [55]. The grain boundary vacancy formation enthalpy $\left( \Delta H_{\square ,f}^{\mathrm{gb}}\right)$ is unknown, but a reasonable approximation is to assume the vacancy formation enthalpy at the grain boundary to be 50% of the activation energy for diffusion. In bcc iron, there is a magnetic effect on the diffusivity, which is represented by the factor $\left( \alpha ^{\mathrm{gb}}\right)$ and the spontaneous magnetisation *s*. The bulk diffusivities and the influences of magnetic ordering on their activation energy for the substitutional elements used in bcc iron are obtained from the manuscript of Versteylen and co-workers [56, and references therein].

**Table 1:** Model parameters for self-healing creep steel, for Fe–Au, Fe–Cu, Fe–Mo, and Fe–W alloys

Variable	Value	Unit	References
$Q_{\square }^{\mathrm{gb}}$	0.58	eV	[55]
$\delta D_{0,\square }^{\mathrm{gb}}$	6.35	10^−15^ m^3^s^−1^	[55]
$\alpha ^{\mathrm{gb}}$	1.28	–	[55]
$Q_{Fe}^{\mathrm{bulk}}$	2.83	eV	[56]
$D_{0,Fe}^{\mathrm{bulk}}$	0.35	10^−4^ m^2^s^−1^	[56]
$\alpha _{Fe}^{\mathrm{bulk}}$	0.16	–	[56]
$Q_{Au}^{\mathrm{bulk}}$	2.37	eV	[56]
$D_{0,Au}^{\mathrm{bulk}}$	0.68	10^−4^ m^2^s^−1^	[56]
$\alpha _{Au}^{\mathrm{bulk}}$	0.10	–	[56]
$Q_{Cu}^{\mathrm{bulk}}$	2.58	eV	[56]
$D_{0,Cu}^{\mathrm{bulk}}$	0.27	10^−4^ m^2^s^−1^	[56]
$\alpha _{Cu}^{\mathrm{bulk}}$	0.10	–	[56]
$Q_{Mo}^{\mathrm{bulk}}$	2.65	eV	[56]
$D_{0,Mo}^{\mathrm{bulk}}$	0.59	10^−4^ m^2^s^−1^	[56]
$\alpha _{Mo}^{\mathrm{bulk}}$	0.10	–	[56]
$Q_{W}^{\mathrm{bulk}}$	2.70	eV	[56]
$D_{0,W}^{\mathrm{bulk}}$	0.26	10^−4^ m^2^s^−1^	[56]
$\alpha _{W}^{\mathrm{bulk}}$	0.10	–	[56]
$\Delta H_{\square ,{\mathrm{form}}}^{\mathrm{gb}}$	0.29	eV	–
$\delta$	0.5	10^−9^ m	–
*d*	30	10^−6^ m	–
*a*	0.5	10^−6^ m	–
$\lambda$	5	10^−6^ m	–
$\varOmega$	11.7	10^−30^ m^3^	[57]

The diffusivity parameters, the vacancy concentrations, the volume of a vacancy, the thickness of a grain boundary, the considered creep cavity radius and spacing, and the applied stress that are used as modelling parameters to obtain the critical stresses for self-healing and the efficiency for self-healing are gathered in Table 1. These model parameters are used to estimate the critical stress for self-healing of diffusional creep damage. For reference, the supersaturations at ${T} = 550~^{\circ }$ and 650° are listed in Table 2.

**Figure 6:**
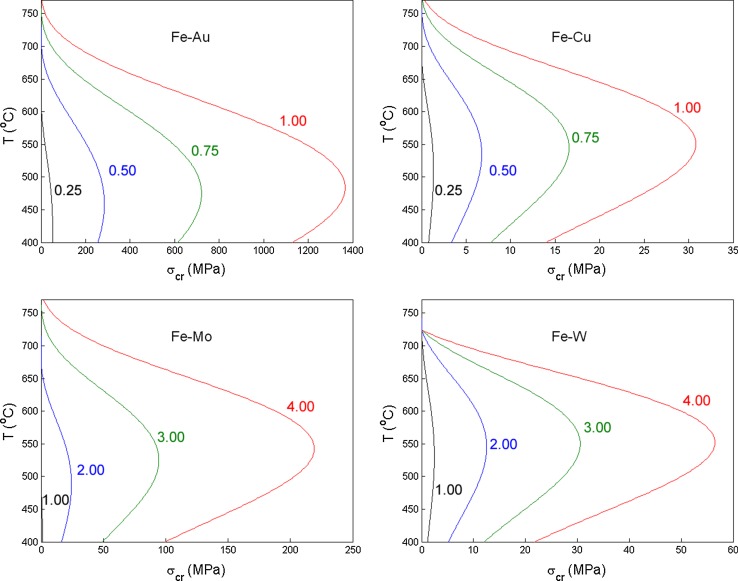
Critical stress $\sigma _{\mathrm{crit}}$ for the self-healing of creep damage as a function of temperature *T* for different compositions of binary Fe–Au, Fe–Cu, Fe–Mo, and Fe–W alloys. The nominal solute concentration (in at.%) is indicated for each curve. Molybdenum (Mo) and tungsten (W) are more soluble at high temperatures and therefore analysed for higher solute contents

The critical stress was calculated for Fe–Au, Fe–Cu, Fe–Mo, and Fe–W alloys for different solute contents (Fig. 6) assuming that all supersaturated solute experiences a driving force for the selective precipitation at the creep cavity surfaces. The self-healing process in Fe–1at.%Au is found to be functional up to relatively high stresses, due to the high diffusivity of Au in the Fe bulk. At high temperatures, the efficiency drops quickly, which is caused by: (1) the decrease in amount of supersaturated solute available for self-healing and (2) the diffusivities of solute and host are getting closer to each other at high temperatures. In addition, the activation energy for grain boundary diffusion shows a considerable temperature evolution close to the Curie temperature [55].

**Table 2:** Supersaturation of different solute containing alloys, at a temperature of 550° and in brackets (650°)

Alloy	Concentration (at.%)	Supersaturation (at.%)
Au	0.25	0.10 (0.00)
	0.50	0.35 (0.19)
	0.75	0.60 (0.44)
	1.00	0.85 (0.69)
Cu	0.25	0.19 (0.09)
	0.50	0.44 (0.34)
	0.75	0.69 (0.59)
	1.00	0.94 (0.84)
Mo	1.00	0.00 (0.00)
	2.00	0.84 (0.25)
	3.00	1.84 (1.25)
	4.00	2.84 (2.25)
W	1.00	0.80 (0.67)
	2.00	1.80 (1.67)
	3.00	2.80 (2.67)
	4.00	3.80 (3.67)

The mechanism that reduces the growth rate of the creep cavities also functions at stresses higher than the previously determined critical stress for self-healing. In this range $\left( \sigma > \sigma _{cr}\right)$, the reduction in creep strain rate can be expressed in a parameter $\eta$;

17 $$\begin{aligned} \eta = 1-\frac{\dot{\epsilon }\left( \Delta x_{\mathrm{sol}}\right) }{\dot{\epsilon }(0)}= \frac{k_{\mathrm{B}} T}{\varOmega \sigma } \frac{4\lambda ^3}{\pi a \delta l} \frac{\left( D_{\mathrm{sol}}^{\mathrm{bulk}}-D_{\mathrm{host}}^{\mathrm{bulk}}\right) \Delta x_{\mathrm{sol}}^{\mathrm{bulk}}}{D_{\square }^{\mathrm{gb}} x_{\square }^{\mathrm{gb}} }. \end{aligned}$$

**Figure 7:**
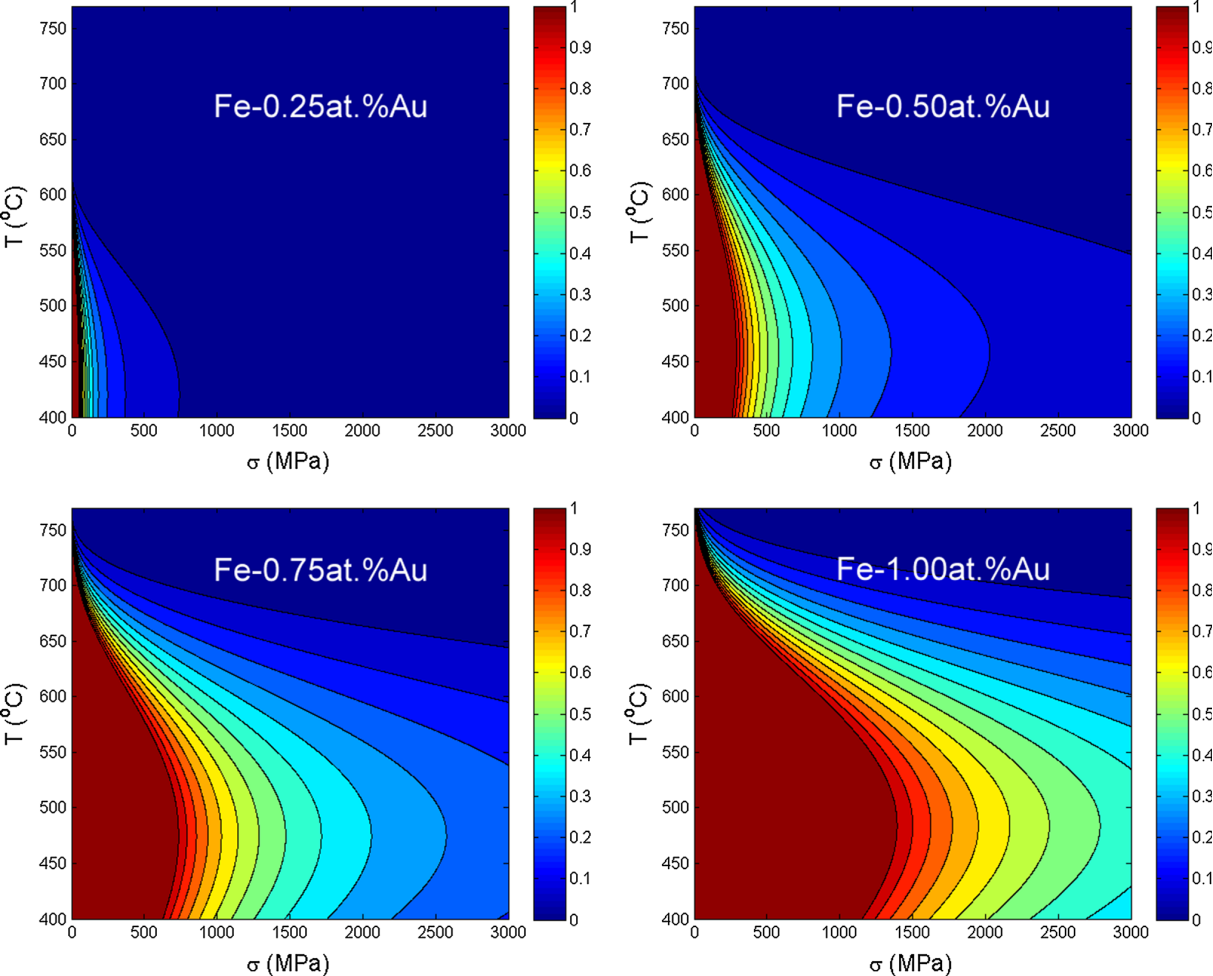
Self-healing efficiency $\eta = 1-\frac{\dot{\epsilon }(\Delta x_{\mathrm{sol}})}{\dot{\epsilon }(0)}$, as a function of stress and temperature. The critical stress for complete self-healing ($\eta =1$) as a function of temperature is indicated by the red line, and partial self-healing ($\eta <1$) is indicated by the other colours. The Fe–Au healing is indicated as a function of concentration between 0.25 and 1% of nominal concentration

**Figure 8:**
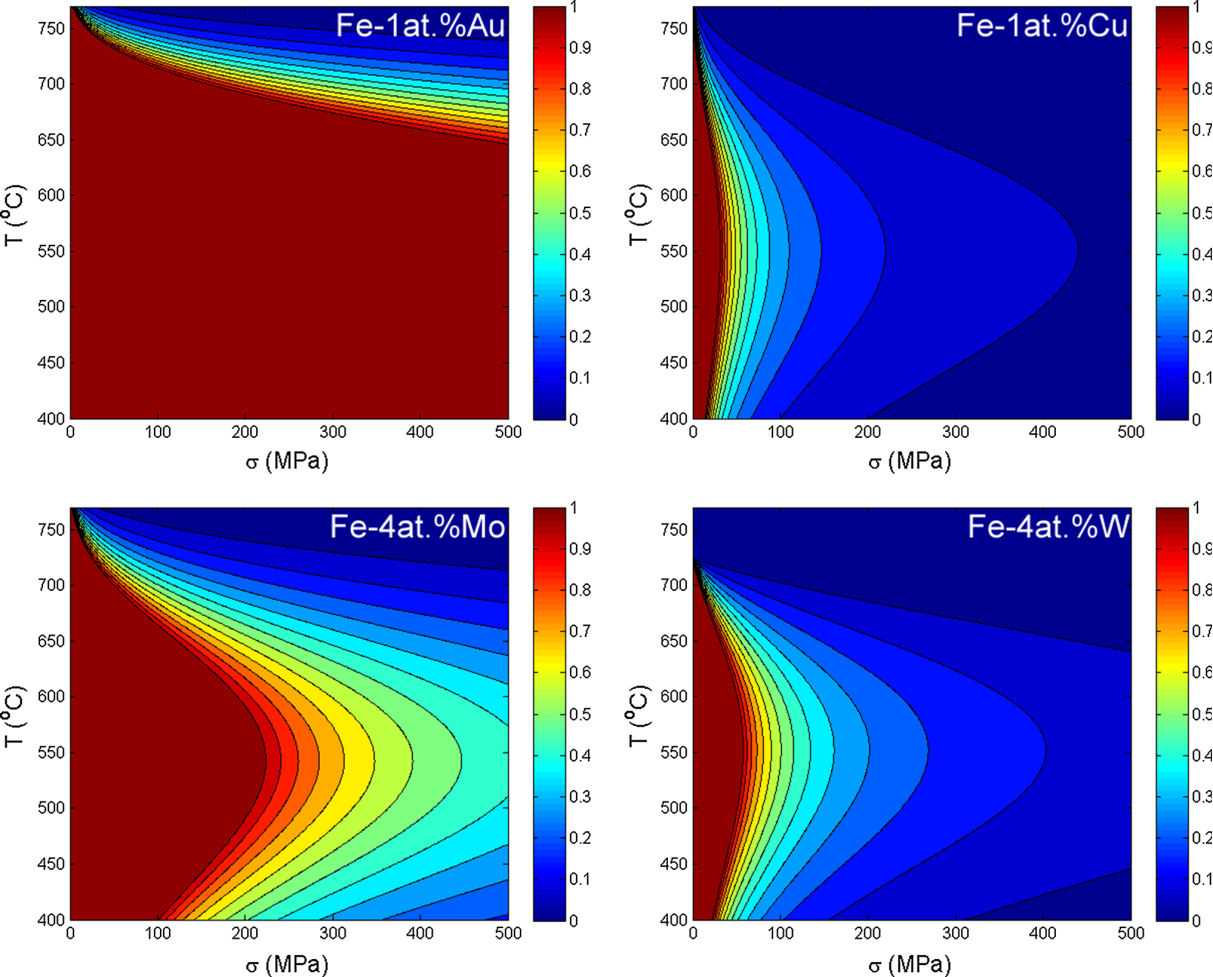
Self-healing efficiency $\eta = 1-\frac{\dot{\epsilon }(\Delta x_{\mathrm{sol}})}{\dot{\epsilon }(0)}$, as a function of stress and temperature. The critical stress for complete self-healing ($\eta =1$) as a function of temperature is indicated by the red line, and partial self-healing ($\eta <1$) is indicated by the other colours. Fe–Cu and Fe–Au contain 1% nominal concentration of solute, and Fe–W and Fe–Mo contain 4% nominal concentration of solute

The efficiency of the self-healing process goes to zero at very high stresses and to $\eta =1$ at the critical stress. In Fig. 7, the efficiency of self-healing is indicated as a function of stress and temperature at different Au concentrations. The addition of molybdenum and tungsten in solid solution is common for creep steels [58] and is generally related to the formation of nanoprecipitates in creep steels. In a recent article by Fedoseeva and co-workers however, it was shown that a commercial alloy with added tungsten content loses creep strength after prolonged creep times, which coincides with the depletion of solute tungsten [59]. When molybdenum or tungsten is added in excess, keeping a percentage in solution, the solubility can extend to high temperatures. The temperature reach for self-healing can therefore be much higher than for copper or gold (see Fig. 5). The efficiency of self-healing is therefore analysed for higher nominal concentrations or molybdenum and tungsten (see Fig. 8).

The efficiency and critical stress for self-healing strongly depend on the relative distance between cavities compared to the size of the creep cavities, $\frac{\lambda }{a}$. The difference in bulk diffusivities between Cu and Au predicts that the self-healing process will work much more efficiently for Fe–Au than for Fe–Cu at the same degree of supersaturation. This is in concurrence with what was found in experiments [60]. As expected, temperature has a large effect on the efficiency of self-healing since the self-healing and damage formation processes are diffusional in nature.

Self-healing behaviour has potentially been observed in Al–Mg alloys as well [61], where voids were filled by segregation of Mg to a void site. Examples for (partial) self-healing of creep damage by precipitation may also have been observed in other aluminium alloys. For instance, Yousefiani and co-workers [62] presented the creep strain rates of overheated aluminum alloys. In their samples, the precipitates of a 7075 aluminum alloy were dissolved at high temperatures, which caused lower creep strain rates, and bulky precipitates on the grain boundaries, which also appear on self-healing creep cavities in Fe–Au and Fe–Mo alloys [25, 26, 29]. In the article of Fedoseeva and co-workers [59], the effect may have been observed in a commercial creep steel, with bulky grain boundary precipitates and an increase in creep rate after the depletion of tungsten from solid solution [59].

## Conclusions

A quantitative model was presented to predict how supersaturated solute can be used to heal creep damage, strongly reduce stage II creep rates, and thereby extend creep lifetimes. This process could be complementary to conventional methods for creep-resistant metals. The creep cavity growth rate and the strain rate in metal alloys are closely linked during steady-state creep. The creep cavities grow through the drainage of vacancies from grain boundaries on which the cavities nucleate. The vacancy formation on the grain boundaries is linked to the rate of ingress of dislocations to that grain boundary. During the selective precipitate growth process in the creep cavities, a transport of solute atoms takes place. The creep cavities grow by a diffusional flux of vacancies, driven by the stress on the grain boundary, which is proportional to the applied stress. This vacancy flux can be countered by a flux of substitutional solute towards creep cavities. The growth of precipitates can thereby reduce growth rates of a creep cavity, reduce the strain rate, and increase the creep lifetime. The self-healing efficiency can be described as a function of the amount of supersaturated solute and the relative diffusivities, assuming selective precipitation at the free creep cavity surfaces. It is found that Au is the most efficient solute element for self-healing of creep damage, and the addition of Au to a creep-resistant steel is thought to have little effect on other precipitates and could be implemented in creep-resistant steels. Mo and W provide a good and low-cost alternative that have potential for self-healing as long as they remain in supersaturation.

## Figures and Tables

**Figure 9:**
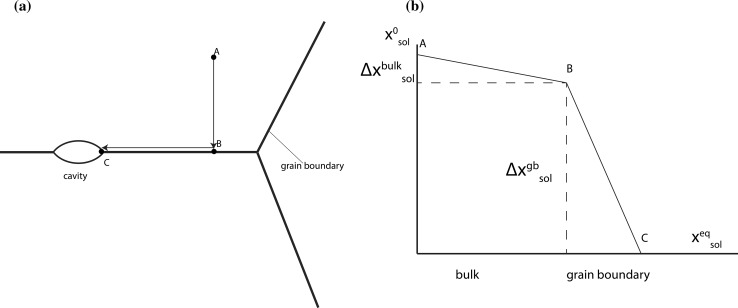
**a** The creep void, which is formed on a grain boundary, acts as a sink for supersaturated solute atoms. **b** The solute concentration at the edge of the creep void is equal to the equilibrium, and far away from the creep void, the supersaturation concentration is maximum. Depending on the diffusivity in the grain boundary and through the bulk, there will be a concentration gradient formed

## Appendix: Flow resistance for solute diffusion

Consider a creep cavity on a grain boundary. The creep cavity acts as a sink for solute atoms, which can diffuse through the bulk and through grain boundaries (see Fig. 9). The supersaturated solute flows towards the sink, with a rate dependent on the diffusivity through the bulk and through the grain boundary. For a more detailed analysis of the relative influence of the grain boundary and bulk diffusivities, the authors would like to refer to earlier work [63]. This problem is analogous to the electric current through two resistors in series. The current depends on both resistors in the same way as the flux towards the creep cavity will depend on the diffusivity the grain boundary and in the bulk.

Starting from the local flux of solute atoms described by Fick’s law,

A.1 $$\begin{aligned} \vec {J}_{\mathrm{sol}} = -\frac{1}{\varOmega }D_{\mathrm{sol}} \vec {\nabla }x_{\mathrm{sol}}. \end{aligned}$$

For a creep cavity, the integrated flux of solute (in number of atoms per unit of time) can be expressed as:

A.2 $$\begin{aligned} I = \frac{\dot{V}}{\varOmega } = \int _A\vec {J}_{\mathrm{sol}} \cdot {\hbox {d}}\vec {S} = \frac{\Delta x_{\mathrm{sol}}}{R}. \end{aligned}$$

For a diffusion length *L*, and cross section *A*, the flow resistance can be estimated with:

A.3 $$\begin{aligned} R = \frac{L}{A D_{\mathrm{sol}}} \end{aligned}$$

Assuming that (1) most of the solute that flows to the creep cavity originates from the bulk (grain boundary volume is low compared to the creep cavity volume) and (2) we only consider the timescales where the bulk diffusion is significant. Under these conditions, we can approximate the diffusional flow of solute atoms as a bulk diffusion and a grain boundary diffusion process in series. A generalisation to other cases was recently presented elsewhere [63].

The flow resistance for the bulk diffusion can be approximated by assuming that the effective cross section equals two times the grain boundary surface available for an individual cavity $A_{\mathrm{bulk}} = 8 \lambda ^2$ (to account for the bulk diffusion from both sides of the grain boundary) and that $L_{\mathrm{bulk}} \approx l= \sqrt{\pi D_{\mathrm{sol}}^{\mathrm{bulk}} t}$ is the diffusion length, resulting in:

A.4 $$\begin{aligned} R_{\mathrm{bulk}} = \frac{L}{A_{\mathrm{bulk}} D_{\mathrm{sol}}^{\mathrm{bulk}}} \approx \frac{l}{8 \lambda ^2 D_{\mathrm{sol}}^{\mathrm{bulk}}}. \end{aligned}$$

The time-dependent diffusion length can be approximated by its maximum value at complete filling of $l_{\mathrm{max}} \approx \pi a^3/3 \lambda ^2 \Delta x_{\mathrm{sol}}$. The flow resistance for the grain boundary diffusion can be approximated by assuming that $A_{\mathrm{gb}}\approx 2\pi a \delta$ and $L_{\mathrm{gb}} \approx \lambda$, resulting in:

A.5 $$\begin{aligned} R_{\mathrm{gb}} = \frac{L}{A_{\mathrm{gb}}D_{\mathrm{sol}}^{\mathrm{gb}}} \approx \frac{\lambda }{2 \pi a \delta D_{\mathrm{sol}}^{\mathrm{gb}}}. \end{aligned}$$

The total flow resistance between the nominal solute concentration in the bulk and the solute at the creep cavity surface then corresponds to:

A.6 $$\begin{aligned} R_{tot} = R_{\mathrm{bulk}} + R_{\mathrm{gb}} \approx \frac{l}{8 \lambda ^2 D_{\mathrm{sol}}^{\mathrm{bulk}}}+\frac{\lambda }{2 \pi a \delta D_{\mathrm{sol}}^{\mathrm{gb}}}. \end{aligned}$$

The total supersaturation of solute is now divided over the bulk and the grain boundary as: $\Delta x_{\mathrm{sol}}^{\mathrm{bulk}} = \Delta x_{\mathrm{sol}} R_{\mathrm{bulk}}/(R_{\mathrm{bulk}} +R_{\mathrm{gb}})$ and $\Delta x_{\mathrm{sol}}^{\mathrm{gb}} = \Delta x_{\mathrm{sol}} R_{\mathrm{gb}}/(R_{\mathrm{bulk}} +R_{\mathrm{gb}})$, respectively.

For $l \approx l_{\mathrm{max}}$, the ratio of bulk and grain boundary flow resistances is:

A.7 $$\begin{aligned} \frac{R_{\mathrm{gb}}}{R_{\mathrm{bulk}}} \approx \frac{a D_{\mathrm{sol}}^{\mathrm{bulk}}}{\delta D_{\mathrm{sol}}^{\mathrm{gb}}} \frac{12 \lambda ^5}{\pi ^2 a^5} \Delta x_{\mathrm{sol}} \ll 1. \end{aligned}$$

For the Fe–Au alloy with $\Delta x_{\mathrm{sol}} = 0.01$, $a= 0.5$ $\mu \mathrm{m}$, $\lambda = 10$ $\mu \mathrm{m}$, $\delta = 0.5$ nm, $D_{\mathrm{sol}}^{\mathrm{bulk}}~=~7.47~\times$ 10^−21^ m^2^s^−1^, and $D_{\mathrm{sol}}^{\mathrm{gb}}~=~7.43~\times$ 10^−12^ m^2^s^−1^ at $T = 823$ K, (Table 1), we find $R_{\mathrm{gb}}/R_{\mathrm{bulk}} = 0.03$ $\ll$ 1. As a result, we can assume $R_{tot} \approx R_{\mathrm{bulk}}$ and $\Delta x_{\mathrm{sol}}^{\mathrm{bulk}} \approx \Delta x_{\mathrm{sol}}$.
